# Artificial Intelligence in Neurosurgery: A State-of-the-Art Review from Past to Future

**DOI:** 10.3390/diagnostics13142429

**Published:** 2023-07-20

**Authors:** Jonathan A. Tangsrivimol, Ethan Schonfeld, Michael Zhang, Anand Veeravagu, Timothy R. Smith, Roger Härtl, Michael T. Lawton, Adham H. El-Sherbini, Daniel M. Prevedello, Benjamin S. Glicksberg, Chayakrit Krittanawong

**Affiliations:** 1 Division of Neurosurgery, Department of Surgery, Chulabhorn Hospital, Chulabhorn Royal Academy, Bangkok 10210, Thailand; j.tangsrivimol@gmail.com; 2 Department of Neurological Surgery, The Ohio State University Wexner Medical Center and Jame Cancer Institute, Columbus, OH 43210, USA; 3 Department Biomedical Informatics, Stanford University School of Medicine, Palo Alto, CA 94305, USA; 4 Department of Neurosurgery, Stanford University School of Medicine, Palo Alto, CA 94305, USA; 5 Stanford Neurosurgical Artificial Intelligence and Machine Learning Laboratory, Department of Neurosurgery, Stanford University School of Medicine, Stanford, CA 94305, USA; 6 Department of Neurosurgery, Computational Neuroscience Outcomes Center (CNOC), Mass General Brigham, Harvard Medical School, Boston, MA 02115, USA; 7 Weill Cornell Medicine Brain and Spine Center, New York, NY 10022, USA; 8 Department of Neurosurgery, Barrow Neurological Institute (BNI), Phoenix, AZ 85013, USA; 9 Faculty of Health Sciences, Queen’s University, Kingston, ON K7L 3N6, Canada; 10 Hasso Plattner Institute for Digital Health, Icahn School of Medicine at Mount Sinai, New York, NY 10029, USA; 11 Cardiology Division, New York University Langone Health, New York University School of Medicine, New York, NY 10016, USA

**Keywords:** artificial intelligence (AI), machine learning (ML), deep learning (DL), artificial Neural Networks (ANN), Convolutional Neural Networks (CNN), Recurrent Neural Networks (RNN), neurosurgery

## Abstract

In recent years, there has been a significant surge in discussions surrounding artificial intelligence (AI), along with a corresponding increase in its practical applications in various facets of everyday life, including the medical industry. Notably, even in the highly specialized realm of neurosurgery, AI has been utilized for differential diagnosis, pre-operative evaluation, and improving surgical precision. Many of these applications have begun to mitigate risks of intraoperative and postoperative complications and post-operative care. This article aims to present an overview of the principal published papers on the significant themes of tumor, spine, epilepsy, and vascular issues, wherein AI has been applied to assess its potential applications within neurosurgery. The method involved identifying high-cited seminal papers using PubMed and Google Scholar, conducting a comprehensive review of various study types, and summarizing machine learning applications to enhance understanding among clinicians for future utilization. Recent studies demonstrate that machine learning (ML) holds significant potential in neuro-oncological care, spine surgery, epilepsy management, and other neurosurgical applications. ML techniques have proven effective in tumor identification, surgical outcomes prediction, seizure outcome prediction, aneurysm prediction, and more, highlighting its broad impact and potential in improving patient management and outcomes in neurosurgery. This review will encompass the current state of research, as well as predictions for the future of AI within neurosurgery.

## 1. Introduction

**Figure 1:**
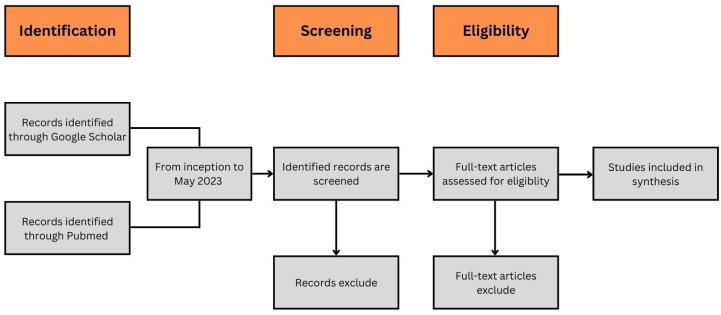
Literature Search Method. Figure Description: PubMed and Google Scholar were searched using AI-related keywords for English literature published from inception to May 2023. Observational studies, case–control studies, cohort studies, clinical trials, meta-analyses, reviews, and guidelines were reviewed.

In the past half-decade, there has been a considerable amount of discourse surrounding the subject of Artificial Intelligence (AI). AI encompasses the utilization of computer systems to accomplish objectives through the simulation of cognitive capabilities [1,2]. Upon conducting a comprehensive review of previously published articles, it is evident that the utilization of AI is progressively becoming more widespread in various biomedical fields. Numerous systematic reviews have been published [3,4,5,6], with the majority attempting to consolidate a vast number of research papers. However, a significant gap exists in the integration of AI with clinical expertise, which is a crucial aspect that needs to be addressed. While some clinicians can comprehend, create, and apply AI techniques, a significant proportion, including neurosurgeons, need more familiarity with this domain. Most clinicians have limited exposure to AI due to their primary concentration on surgical practice and the era of their training. Hence, this paper aims to identify high-cited seminal papers, as higher citation rates indicate clinician engagement, and to summarize and highlight the various applications of machine learning. Such an endeavor aims to enhance understanding among clinicians, paving the way for future utilization as familiarity grows over time. The literature search method involved searching PubMed and Google Scholar using relevant keywords to identify English literature publications from its inception until May 2023. A comprehensive review was performed on various studies, including observational studies, case–control studies, cohort studies, clinical trials, meta-analyses, reviews, and guidelines, as depicted in Figure 1. For ease of navigation and clarity, the information will be presented in four categories: tumor, spine, functional, and vascular. This wide range of topics will be examined to obtain a comprehensive understanding of the advancements and challenges associated with integrating AI in these domains.

**Figure 2:**
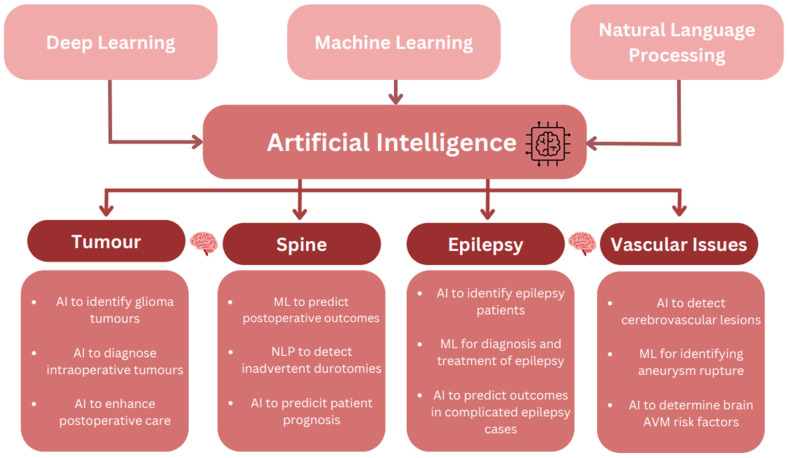
Summarizes potentials of AI in neurosurgery.

To obtain a comprehensive understanding of the integration of AI in medicine, it is essential to first differentiate between AI, machine learning (ML), and deep learning (DL). While the idea of AI has existed for a considerable length of time, ML is a subfield of AI which seeks to learn patterns from data [7,8,9] that can be broadly divided into two categories: supervised learning and unsupervised learning [10]. However, a third category of weakly supervised learning and/or reinforcement learning may be considered given its importance in modern real-world ML (e.g., Chat GPT). Supervised learning involves creating predictions based on preliminary data or data groups with labeled outcomes, whereas unsupervised learning does not learn from or have access to labeled outcomes [10]. While both types of learning can be employed to create quantitative predictions, unsupervised learning can uncover new classification or patterns. Here, we review all emerging applications of these machine learning technologies in neurosurgery (Figure 2).

**Figure 3:**
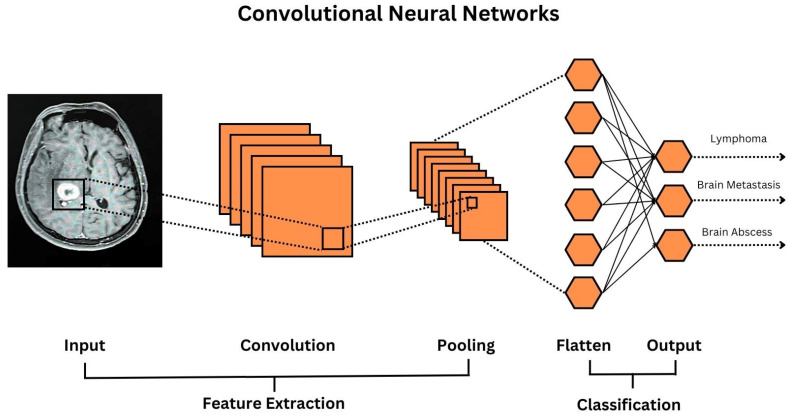
Convolutional neural network.

Machine learning forms the foundation of DL [11,12], which employs Artificial Neural Networks (ANN) designed to mimic cognitive brain function to learn complex patterns in data. Computer vision tasks often utilize Convolutional Neural Networks (CNN) which can learn and identify visual patterns [13] (Figure 3). Natural Language Processing Tasks have historically used Recurrent Neural Networks (RNN), which can encode time/sequence-based information, such as in language, but is now primarily transformer-based (e.g., Chat GPT, pretrained medical large language models [GatorTron], etc.…) [14,15]. Clinicians must educate themselves on the various forms of AI, as their understanding of these technologies (and not blind trust) is essential in ensuring its proper and safe translation for patient care.

## 2. Tumor

AI has been applied for the brain tumor classification task [16,17,18,19,20,21]. In a recent Buchlak et al. investigation, AI was leveraged for identifying and categorizing glioma tumors through neuroimaging analysis [22]. The researchers systematically evaluated 153 studies that employed ML [CNN, Support Vector Machine (SVM),Random Forest (RF)] to enhance tumor grading, diagnosis, segmentation, non-invasive genetic biomarker identification, progression monitoring, and patient survival prognosis. In general, the performance of the ML model was excellent (AUC = 0.87 ± 0.09; sensitivity = 0.87± 0.10; specificity = 0.86 ± 0.10; precision = 0.88 ± 0.11). Their findings revealed that CNN, SVM, RF demonstrated the most favorable outcomes. This investigation underscores ML’s critical role in medical classification and its potential to significantly enhance disease diagnosis and treatment. This highlights the limitations of a review on machine learning applications to glioma MRI data, including the influence of a large sample size on NLP classification models and the exclusion of conference papers, while suggesting the use of optimized deep language models and referring readers to specific papers for further information.

Distinguishing between primary CNS lymphoma (PCNSL) and glioblastoma multiforme (GBM) based on MRI findings can be challenging. McAvoy et al. employed the EfficientNetB4 architecture within a convolutional neural network (CNN) framework to analyze contrast-enhanced T1-weighted images from a cohort of 320 patients with suspected GBM or PCNSL [23]. The findings demonstrated that CNN-based analysis could effectively assist radiologists in achieving accurate differential diagnoses between these two entities (mean 5-fold cross-validation AUC = 0.71). This research highlights the potential of CNNs as a valuable tool in aiding the diagnostic process and improving the precision of differential diagnosis for PCNSL and GBM based on MRI imaging. This paper aims to assist physicians in formulating a comprehensive and accurate differential diagnosis, ultimately leading to faster and more appropriate treatment measures. This study has limitations, including its retrospective design with a small number of patients from two academic institutions, which may limit the generalizability of the findings to other settings, the use of PNG exports of DICOM images leading to loss of data, and the absence of a direct comparison between the classification outcomes of CNNs and radiologists, thus requiring further research to determine the tool’s clinical value.

Accurate cortical segmentation and volume assessment play a vital role in the continuous surgical planning of patients and monitoring of treatment response. To address this task, Boaro et al. employed a 3D convolutional neural network (3D-CNN) to achieve expert-level automated segmentation and volume estimation of meningiomas from MRI scans [24]. An initial training phase involved training a 3D-CNN by segmenting complete brain volumes utilizing a dataset comprising 10,099 MRIs of healthy brains. Subsequently, through the implementation of transfer learning, the network underwent specific training for meningioma segmentation, using a dataset consisting of 806 labeled MRIs. Their approach yielded an impressive accuracy of 88.2%. This highlights the potential of their method for precise and reliable cortical segmentation, enabling enhanced patient care and treatment evaluation. The limitations of this study include the inability to evaluate post-operative residuals, tumor recurrence, or tumor growth, due to the inclusion of single pre-operative scans, the lack of testing the model on brain MRI scans without meningioma for assessing detection performance, the absence of integrating the algorithm into the hospital informatics system, and the retrospective nature of the study requiring prospective validation for real-world clinical applicability.

ML may also be used to identify and characterize predictive variables for treatment prognosis. The predictive value of isocitrate dehydrogenase (IDH) mutation status and 1p19q codeletion, as indicators of treatment response in glioma, remains an area of interest. Zhou et al. focused on the predictive value of isocitrate dehydrogenase (IDH) mutation status and 1p19q codeletion as indicators of treatment response in glioma from MRI patterns [25]. The training cohort consisted of preoperative MRIs from 538 glioma patients spanning three different institutions. They utilized a random forest algorithm to construct a predictive model that classified gliomas into three categories: IDH-wildtype, IDH-mutant with 1p19q codeletion, and IDH-mutant without 1p19q codeletion. The study results showed a successful prediction rate of 78.2%, accurately classifying 155 out of 198 cases. The performance of IDH was evaluated by calculating the area under the receiver operating characteristic curve (AUC), which yielded a value of 0.921. Similarly, in the validation cohort, IDH achieved an AUC of 0.919. Age provided the greatest predictive value, followed by shape features. These findings highlight the potential of the developed model in accurately determining the IDH mutation status and 1p19q codeletion status from MRI patterns in glioma patients. The study’s limitations include its retrospective design with a focus on known gliomas, limiting the model’s applicability to situations with different tumor types and non-tumor mimickers, and the need for a more general model using data from other lesion types to improve generalizability. Furthermore, the study did not incorporate advanced MR modalities, such as perfusion MRI and MR-spectroscopy, which could enhance the prediction of IDH genotype.

U-Net is an artificial neural network architecture primarily used for image segmentation in computer vision. Its U-shaped structure consists of an encoding path that captures features through convolutional layers and downsampling, and a decoding path that recovers spatial resolution using upsampling and skip connections. These skip connections fuse low-level and high-level features to capture fine details and context. The output is a segmentation mask with class labels for each pixel. U-Net has proven highly effective in medical imaging, but can also be applied to other segmentation tasks. The BraTS (Brain Tumor Segmentation) dataset is a widely used benchmark dataset in medical imaging for brain tumor segmentation. It contains multi-modal MRI scans of patients with annotated tumor regions. The dataset serves as a standard for developing and evaluating algorithms for automatic tumor segmentation, enabling researchers to compare their methods and advance the field. BraTS has been instrumental in the development of accurate and efficient brain tumor segmentation techniques. Huang et al. and Yousef et al. address the challenges associated with the segmentation of brain tumors from MRI scans [26,27]. Huang et al. propose a deep multi-task learning framework incorporating a multi-depth fusion module and a distance transform decoder to achieve accurate segmentation [26]. On the other hand, Yousef et al. highlight the prevalence of U-Net-based models in medical imaging [27]. They evaluate various variants of U-Net and emphasize the importance of developing new architectures to optimize medical image analysis. The aforementioned paper proves highly valuable in the context of brain tumor classification. However, more emphasis should be placed on discussing the architectural aspects of the deep machine learning utilized. Instead, this paper primarily focuses on clinical knowledge.

AI has also been implemented in surgical procedures to assist in the planning phase, aiding in decision-making during surgery. Its utilization has shown promising results, enhancing the accuracy of surgical positioning and mitigating surgical complications [28,29,30,31,32]. In a recent investigation by Tonutti et al., ML was employed to facilitate the diagnosis of intraoperative tumors [32]. Specifically, the researchers used ML algorithms, such as ANNs and Support Vector Machine (SVM), to develop personalized anatomical models for intra-operative use. Integrating augmented reality (AR) with these models further enhanced surgical precision. The results revealed that SVR yielded more precise outcomes than ANN, with positional errors of less than 0.2 mm. Furthermore, the model was observed to be more accurate and personalized than real-time deformation models, thereby illustrating its potential to revolutionize the field of surgery. The study highlights assumptions and simplifications in the development of a biomechanical brain model for machine learning, including the use of generic mechanical parameters, exclusion of certain brain structures, and limitations in accounting for topological changes during surgery, suggesting the need for more advanced simulations and real-time imaging to make the method applicable in clinical settings.

Shen et al. have developed a pioneering approach for the intraoperative diagnosis of glioma utilizing deep CNNs and fluorescence imaging (FL-CNN) [33]. A total of 23 patients diagnosed with glioma participated in the study, wherein they underwent fluorescence image-guided surgery after receiving injections of indocyanine green. Following the surgical procedures conducted on these patients, 1874 tissue samples were carefully collected. Additionally, fluorescence images in the second near-infrared window (NIR-II, 1000–1700 nm) were acquired to provide detailed visual information. A FL-CNN was utilized for automated glioma diagnosis and compared to the gold-standard pathology for intraoperative diagnosis. The study revealed that FL-CNN exhibited superior sensitivity (93.8% vs. 82.0%, *p* < 0.001) and specificity of over 80%, without any additional time, outperforming neurosurgeons. Moreover, the FL-CNN effectively corrected nearly 70% of neurosurgeon errors. Additionally, the FL-CNN could predict tumor grade and Ki67 with AUCs of 0.81 and 0.625, respectively. These findings demonstrate that FL-CNNs are more effective than neurosurgeons, making them suitable for intraoperative glioma diagnosis. The limitation of the FL-CNN approach include its reliance on NIR-II fluorescence imaging, which offers advantages over NIR-I but may still exhibit lower specificity compared to clinically available 5-ALA fluorescence. However, the FL-CNN demonstrates comparable specificity and higher sensitivity than 5-ALA when equipped with deep learning, and the experiment results were confirmed by pathological examination using a gold standard, ensuring precise performance measurement and objective comparison with neurosurgeons.

Hollon et al. conducted a similar investigation to assess the efficacy of Raman-based imaging, coupled with CNNs, compared to board-certified neuropathologists for diagnosing glioma molecular class identification [34]. Inputs to the model included: Raman spectroscopy derived imaging, coherent anti-Stokes Raman scattering (CARS) microscopy, and stimulated Raman histology. By employing a boosted tree algorithm to classify intraoperative Raman spectra, they could discern normal brain tissue from areas invaded by tumors, specifically those with a tumor cell invasion exceeding 15%. This classification approach yielded a remarkable accuracy rate of 92% (with a sensitivity of 93% and specificity of 91%). This imaging technique enables the analysis of specimens down to the molecular level and has a sub-micron resolution, providing highly detailed information. This advance can enable subsequent CNN to be applied beyond the details and patterns discernible to the human eye.

AI has been developed to predict outcomes following brain tumor surgery [35,36,37,38,39]. Given the increasing lifespan of patients with brain metastasis and the rising incidence of leptomeningeal disease (LMD), studies on LMD as a risk factor are limited. Tewarie et al. examined leptomeningeal disease (LMD) as a risk factor in brain metastasis patients’ rising lifespans [40]. They used the conditional survival forest, Cox proportional hazards model, XGBoost classifier, extra trees classifier, logistic regression, and SMOTE to overcome class imbalance. In 168 (15.9%) of 1054 brain metastasis patients who had surgery, LMD occurred at a median time of 7.05 months following diagnosis. For the optimal Leptomeningeal Disease (LMD) occurrence discrimination, utilizing an XGboost algorithm proved highly effective, resulting in an impressive AUC of 0.83. Furthermore, when it came to prognosticating the time until LMD development, the random forest algorithm and the Cox proportional hazards model exhibited comparable performance, with a concordance index (C-index) of 0.76. Notably, proximity of brain metastasis to the cerebrospinal fluid space and the site of cerebellar brain metastasis were important factors in both LMD classification and regression. In addition, lymph node metastasis of the primary tumor at the time of brain metastasis diagnosis emerged as a significant risk factor influencing both the incidence of LMD and the time to LMD. The limitations of the study include the wide time span during which patients were included, the classification of radiographic elements based on clinical relevance, the need for further research on the isolated role of radiographic components in LMD, the novelty of lymph node metastasis as an LMD risk factor, the exclusion of patients receiving only radiation therapy, the reduced variability of the data due to the use of SMOTE, the theoretical nature of LMD prognostication at BM diagnosis in clinical care, the need for external validation of the models, and the exploration of possible novel LMD risk factors.

Currently, the prediction of brain metastasis (BM) often based on radiotherapy. [41,42,43]. Hulsbergen et al. sought to develop a predictive model for estimating 6-month survival after surgical resection of brain metastasis [44]. The current reliance on radiotherapy-based approaches for brain metastasis prediction prompted the need for an alternative approach. The study utilized an institutional database of 1062 patients and tested seven distinct ML models, with model performance assessed by AUC. The results indicated that logistic regression outperformed other methods, achieving an AUC of 0.71. In comparison, the diagnosis-specific graded prognostic assessment achieved an AUC of 0.66. These findings suggest that the developed model holds promise in accurately predicting 6-month survival (*p* < 0.0005) following neurological resection of brain metastasis, facilitating meaningful risk stratification in clinical practice. This study has limitations, including internal validation using retrospective data, the need for external validation in a prospective setting, the focus on survival at a 6-month cutoff rather than overall median survival, the influence of intraoperative and postoperative factors on survival prediction, and the importance of randomized trials for surgical decision making. The model can estimate risk and outcomes for patients undergoing surgery but should not be used to determine whether surgery is appropriate. Further analysis of predictive variables in the model can enable further efforts to improve upon the achieved performance.

Given the significant hurdles encountered in accurately predicting individual patient survival, particularly in glioma [45,46,47,48], Senders et al. aimed to address the difficulty in predicting survival in glioblastoma multiforme (GBM) patients by comparing multiple machine and statistical learning algorithms [49]. They developed an online survival calculator using a training dataset of GBM patients diagnosed between 2005 and 2015. Using 15 statistical and machine learning models (e.g., AFT, bagged decision trees, boosted decision trees, boosted decision trees survival,) trained on demographic, socioeconomic, clinical, and radiologic characteristics, they predicted one-year survival and generated individualized survival curves. The study included 20,821 patients who met the criteria, and the AFT model exhibited superior consistency with a concordance index of 0.70. These findings emphasize the need for analyzing assessment in developing and utilizing survival strategies, highlighting the potential of advanced analytical approaches for improved survival predictions in GBM patients. The limitations of many machine learning algorithms include their restriction to continuous and binary models, the inability to compute subject-level survival curves, lack of interpretability, computational inefficiency, and the need for evaluating models based on multiple criteria rather than solely prediction performance, as factors unrelated to prediction performance can exclude high-performing models from clinical deployment. Furthermore, the predictive performance can vary depending on the number and nature of input features, such as the inclusion of multimodal data like radiogenomics.

The evaluation of treatment response for glioma frequently necessitates the utilization of MRI imaging techniques such as MR perfusion and diffusion tensor imaging (DTI) [43,50,51]. In an effort to enhance this process, Chang et al. aimed to improve the evaluation of glioma treatment response using machine learning models with MRI input [52]. They focused on analyzing the hyperintensity of fluid-attenuated inversion recovery (FLAIR), the contrast-enhancing tumor region, and determining tumor volume based on the Neuro-Oncology (RANO) response assessment criteria. Two distinct patient cohorts were employed. The first cohort comprised 843 preoperative MRIs from 843 patients diagnosed with low- or high-grade gliomas originating from four different institutions. The second cohort encompassed 713 longitudinal postoperative MRI visits from 54 patients newly diagnosed with glioblastomas. Each patient in the second cohort had two “baseline” MRIs conducted before the initiation of treatment. It is important to note that this second cohort was exclusively derived from a single institution. In the cohort of postoperative GBM patients, the automatically generated FLAIR hyperintensity volume, contrast-enhancing tumor volume, and AutoRANO were highly repeatable for double-baseline visits, with ICCs of 0.986, 0.991, and 0.977, respectively. Preoperative FLAIR hyperintensity, postoperative FLAIR, and postoperative contrast-enhancing tumor volumes had ICC values of 0.915, 0.924, and 0.965, respectively. Finally, FLAIR hyperintensity volume, contrast-enhancing tumor volume, and RANO measurements had ICCs of 0.917, 0.966, and 0.850 for comparing manually and automatically calculated longitudinal tumor burden changes. AutoRANO, showed promising results in accurately assessing tumors following treatment. These trials will provide valuable insights into the reliability and effectiveness of automated methods for assessing glioma treatment response. The study has several limitations: reliance on segmentations from a single rater, a small patient cohort from a single institution, lack of comparison with other approaches, exclusion of smaller tumors, variability in MR imaging availability, absence of confidence assessment in segmentations, and the need for further validation in larger cohorts and clinical trials.

Applications of AI to the management of neurosurgical care is quickly growing [53,54,55,56]. Senders et al. conducted a study using ML techniques such as ANN, SVM, Fuzzy c-Means (FCM), Bayesian Learning (BL), RF, Logistic regression (LR), Linear Discriminant Analysis (LDA) and others [53]. As of 1 January 2017, over 200 studies of 6,402 citations were identified, involving presurgical planning, intraoperative guidance, neurophysiological monitoring, and neurosurgical outcome prediction. Such works continue to underscore the breadth to which ML can make an impact within neuro-oncological care. The study highlights the limitations of the systematic review, including the need for more detailed analysis of all studies, focus on perioperative care applications, and caution in interpreting the quantitative performance summary. However, it emphasizes that these limitations are proportional to the study’s strengths. It concludes that the review provides an overview of the neurosurgical literature on machine learning and insights into its future direction in the field.

### Current Challenges

Several recent studies have highlighted the potential of machine learning (ML) models in various aspects of neuro-oncological care. Buchlak et al. demonstrated the excellent performance of ML, specifically CNN, SVM, and RF models, in identifying and categorizing glioma tumors through neuroimaging analysis [22]. McAvoy et al. utilized CNN-based analysis to aid radiologists in accurate differential diagnoses between primary CNS lymphoma (PCNSL) and glioblastoma multiforme (GBM) [23]. Boaro et al. achieved high accuracy in cortical segmentation and volume estimation of meningiomas using a 3D-CNN [24]. Zhou et al. developed a predictive model for glioma classification based on IDH mutation status and 1p19q codeletion using a random forest algorithm [25]. Tonutti et al. employed ML algorithms to develop personalized anatomical models for intraoperative tumor diagnosis [32]. Shen et al. utilized deep CNNs and fluorescence imaging for intraoperative glioma diagnosis, outperforming neurosurgeons [33]. Hollon et al. achieved high-glioma molecular class identification accuracy using Raman-based imaging coupled with CNNs [34]. Using various ML algorithms, Tewarie et al. examined the risk factors for leptomeningeal disease (LMD) in brain metastasis patients [40]. Hulsbergen et al. developed a predictive model for estimating 6-month survival after surgical resection of brain metastasis [44]. Senders et al. developed an online survival calculator and conducted a comprehensive review of ML techniques in neuro-oncological care, highlighting their broad impact in different areas of patient management [49,53]. The limitations include small sample sizes, retrospective designs, lack of external validation, and the need for further research and validation in clinical settings. Despite these limitations, machine learning shows promise in improving neurosurgical care, but practical and ethical considerations need to be addressed during implementation.

The potential of AI to revolutionize tumor classification beyond glioblastoma (GBM) and lymphoma is evident, with the ability to discover marker and prognostic genes across various tumor types. These applications could significantly shape the trajectory of treatment. Furthermore, integrating pre-surgical modeling into surgical planning promises to reduce complications due to anatomical variations. AI has shown efficacy in monitoring tumor recurrence in postoperative care, yet the patient’s observations remain crucial. The prospect of leveraging ChatGPT to provide patients with knowledge and alleviate anxiety is feasible, but adequate training and reliable references are vital prerequisites for its application.

## 3. Spine

ML has brought about significant innovation in spine surgery, as evidenced by the consistently high accuracy of outcome data. Most studies in this domain employ machine learning techniques, including ANN, SVM, RF, and others [57,58,59,60,61]. Nida Fatima et al. conducted an ML study to determine the 30-day adverse event rate in patients with Lumbar Degenerative Spondylolisthesis (LDS) following surgery [62]. The study encompassed a dataset of 80,610 patients who underwent LDS surgery, of whom 3965 (4.9%) experienced adverse events within the 30-day postoperative period. ML models, specifically logistic regression and LASSO, were employed to develop 26 prospective models. The final ML algorithms identified several predictors, including gender, age, American Society of Anesthesiologist grade, autogenous iliac bone graft, instrument fusion, surgery levels, surgical approach, functional status, preoperative serum albumin levels (g/dL), and serum alkaline phosphatase levels (IU/mL). Logistic regression consistently demonstrated superior performance in AUC compared to LASSO across various models. This study highlights the potential of utilizing ML techniques in predicting outcomes when dealing with large datasets. Such predictive capabilities can greatly assist in patient counseling and surgical risk assessment. The study has several limitations, including the variation in patient and surgical characteristics within the database used, limited postoperative outcome data beyond 30 days, suboptimal performance of the prediction model with an AUC below 0.8, potential missing variables, coding errors in the data, and the LASSO regression not demonstrating an improved performance compared to logistic regression. Further investigation is needed using alternative machine learning algorithms and larger datasets to enhance predictive accuracy for postoperative adverse events after spinal surgery.

The same assessment can be performed for different outcome statistics. In Karhade et al.’s study, the authors predicted 30-day mortality following spine metastasis surgery from a cohort of 1790 patients [63]. Using various ML algorithms (e.g., Neural Network, Support Vector Machine, Bayes Point Machine, and Decision Tree models), a Neural Network with a c-statistic of 0.769 was the best model for predicting 30-day mortality. They successfully identified preoperative prognostic markers, such as albumin, functional status, WBC, Hct, alkaline phosphatase, spinal location, and concomitant systemic disease. The implication is that these ML algorithms could, preoperatively, accurately predict postoperative outcomes. The study has several limitations, including the variation in patient and surgical characteristics within the database used, limited postoperative outcome data beyond 30 days, suboptimal performance of the prediction model with an AUC below 0.8, potential missing variables, coding errors in the data, and the LASSO regression not demonstrating improved performance compared to logistic regression. Further investigation is needed using alternative machine learning algorithms and larger datasets to enhance predictive accuracy for postoperative adverse events after spinal surgery.

Similarly, Ames et al. conducted an unsupervised AI study to identify surgical factors that predict surgical outcomes in adult spinal deformity (ASD) [64]. The study involved 570 patients divided into three groups: young patients with coronal deformity (*n* = 195), older patients with a history of spinal surgery (*n* = 157), and older patients without prior surgeries (*n* = 218). Hierarchical clustering was employed as the primary methodology to generate representative clusters of patients, characterized by high within-group similarity and the greatest dissimilarity when compared to other groups. Patients were also categorized into 12 groups based on osteotomy type, instrumentation, and interbody fusion. Ultimately, the factors identified allowed clinical reasoning of which patients would undergo surgery with minimal risk. The study has limitations, including the dependency on sample size and observation heterogeneity for determining patient and operative clusters, the potential for further iterative refinements of the model and classification with future data, and the need for additional research to test hypotheses and compare patient-reported outcomes with objective measures.

To evaluate the risk of Adjacent Segment Disease (ASD), it is crucial to consider patients who have undergone previous Anterior Cervical Discectomy and Fusion (ACDF) for cervical reticulopathy, as they are more prone to the occurrence of this condition [65,66,67,68,69]. Goedmakers et al. aimed to predict the development of adjacent segment disease (ASD) in patients undergoing surgery for cervical radiculopathy using DL techniques and preoperative MRI data [70]. They developed a DL model with 48 convolutional layers trained on preoperative T2 sagittal cervical MRI images. The study included 344 eligible patients, of whom 60% (*n* = 208) were used for training, and 40% for validation (*n* = 43) and testing (*n* = 93). The results demonstrated that the deep learning model outperformed assessments made by neuroradiologists and neurosurgeons. The DL model achieved an accuracy of 95%, sensitivity of 80%, and specificity of 97%, whereas the other evaluators had lower accuracy rates of 58%. These findings suggest the potential of DL algorithms to improve the accuracy of ASD prediction based on preoperative MRI data in patients ACDF surgery for cervical radiculopathy. The study had limitations, including reliance on the last available follow-up, lack of consideration for clinical and demographic characteristics, variability in surgical techniques and outcomes, small number of MRI scans, imbalanced distribution of ASD cases, and potential limitations of GradCAM saliency maps.

Karhade et al. specifically applied NLP to operative notes to determine if such analyses could effectively process the large and free-text inputs in our medical record systems, beginning with the operative notes [71,72]. In a cohort of 1000 patients, NLP identified 93 inadvertent durotomies with an AUC of 0.99 [71]. Within the testing set, the NLP algorithm exhibited an impressive sensitivity of 0.89, successfully detecting 16 out of 18 patients who had incidental durotomy. The study has several limitations, including its retrospective design within a single healthcare system, the influence of shared surgical practices on documentation, the lack of prospective and external validation, the potential for unrecognized or unrecorded incidental durotomies, and the impracticality of multiple reviews by different researchers or spine surgeons. The same group used NLP to retrospectively understand the risk factors associated with intraoperative vascular injuries from operative notes [72]. The study found that body mass index, diabetes, L4-L5 exposure, and infection-related surgery (discitis, osteomyelitis) were the best predictors. NLP had a sensitivity of 0.92 when identifying VI from operative notes. Moreover, the algorithm successfully identified 18 out of the 21 patients with VI, resulting in a sensitivity of 0.86. Thus, neurosurgical documentation may be developed as an input to ML models. The study has multiple limitations, including its retrospective design limited to a single healthcare entity, the necessity for prospective validation across multiple institutions, the absence of a well-established gold standard for intraoperative vascular injury, the potential overfitting of the NLP algorithm, the possibility of enhancing performance through collaborative efforts and alternative machine learning-based NLP approaches, and the potential influence of changes in coding practices on the algorithm’s accuracy. Future research could consider comparing institutional records with national databases to evaluate the algorithm’s ability to capture adverse events.

Postoperative opioid prolonged use is a significant concern following spine surgery. Since there has been a noted rise in complications associated with opioids, [73,74,75,76,77,78] Karhade et al. employed five predictive models, namely elastic-net penalized logistic regression, random forest, stochastic gradient boosting, neural network, and the support vector machine, to construct models for predicting prolonged opioid prescriptions [79]. A total of 5413 patients were identified, among whom 416 individuals (7.7%) maintained a prescription for opioid medication between 90 and 180 days following their surgical procedures. The elastic-net penalized logistic regression model had the best discrimination (c-statistic 0.81) and good calibration and overall performance. The investigation revealed that preoperative prediction of prolonged postoperative opioid prescription could enhance surveillance and monitoring post-surgery. Notably, the three most influential predictors in the models were instrumentation, duration of preoperative opioid prescription, and comorbidity of depression. These findings underscore the potential of preoperative prediction in improving the management of postoperative opioid use in patients undergoing spinal surgery. The study acknowledges several limitations, including the unavailability of opioid dose data, the exclusion of illicit opioid use, approximation of opioid use based on medical record data, lack of patient-reported outcomes, limited applicability to other spinal conditions, potential influence of changing surgical techniques over the study period, limited diversity of institutions, and the need for external validation. The web application provided offers opportunities for further evaluation of the models.

ML has also become popular in predicting the accuracy of discharge timelines to improve hospital census. In their study, Stopa et al. investigated the factors influencing delayed discharge after elective spine surgery [80]. A cohort of 144 patients underwent elective inpatient surgery to address lumbar disc disorders, and among them, the rate of nonroutine discharge stood at 6.9% (*n* = 10). The parameters analyzed included age, sex, BMI, ASA class, preoperative functional status, number of fusion levels, comorbidities, preoperative laboratory findings, and discharge disposition. Performance metrics included: discrimination (c-statistic), calibration, and positive and negative predictive values. When applied to the institutional data, the neural network algorithm demonstrated excellent generalization capabilities, as indicated by a c-statistic (AUC) of 0.89. Moreover, the algorithm exhibited a calibration slope of 1.09 and a calibration intercept of −0.08, further attesting to its reliability. Regarding predictive accuracy, at a threshold of 0.25, the positive predictive value (PPV) reached 0.50, while the negative predictive value (NPV) stood impressively at 0.97. Despite the positive findings of this external validation study, it is important to consider other predictive algorithms developed for this population, as they have demonstrated varying levels of performance in terms of discrimination and calibration, highlighting the need for further research and direct comparisons between algorithms in prospective samples. This application to the delayed discharge prediction highlights the applicability of ML to all stages of a patient’s healthcare interaction, where hospitals can translate ML to an array of stages.

For the continuation or follow-up of the treatment plan, it is crucial to identify the brand of the instrument fusion specifically. Huang et al. utilized ML techniques to classify anterior cervical fusion systems, achieving a remarkable accuracy rate of 91.5% ± 3.8% across at least nine distinct types [81]. When considering the top three classifications, the accuracy increased to 98.4% ± 1.3%. These findings demonstrate the potential of machine learning algorithms in conjunction with clinical information to accurately classify anterior cervical fusion systems. The limitations of the current model include the limited number of hardware systems available for training, the need for additional datasets to evaluate visual artifacts and overlapping radiopaque “noise”, the requirement for prospective data to assess clinical utility, and the potential applications of hardware classification beyond revision ACDF surgery. This paper proves to be highly beneficial in situations where patients undergo fixation treatment and experience difficulty recalling the specific brand of rod and screw. Notably, its accuracy in such cases is notably commendable.

### Current Challenges

Using machine learning algorithms, Fatima et al. developed a predictive model for adverse events after lumbar degenerative spondylolisthesis surgery [62]. Logistic regression outperformed LASSO methods, and a web application was created for risk assessment. The study acknowledges limitations such as data variation, limited postoperative outcome data, suboptimal model performance, potential missing variables, and coding errors. Karhade et al. achieved promising results in predicting short-term mortality in spinal metastatic disease using machine learning algorithms and an open access web application [63]. Limitations include data veracity and completeness, limited predictors, inability to capture overall disease trajectory, and need for further evaluation. Ames et al. applied AI-based clustering to classify ASD surgery, but limitations include reliance on radiographic parameters, manual segregation challenges, and need for validation [64]. Goedmakers et al. demonstrated that a deep learning algorithm outperformed experts in predicting adjacent segment disease [70]. Limitations included limited follow-up, demographic considerations, surgical variability, small sample size, imbalanced distribution, and limitations of saliency maps. Karhade et al. used NLP algorithms to detect incidental durotomy, but further validation is needed [71]. Another study by Karhade et al. developed algorithms for detecting intraoperative vascular injury during lumbar spine surgery, but external and prospective validation is necessary [72]. Karhade et al. also created prediction algorithms for prolonged opioid prescription after lumbar disc herniation surgery, with limitations including missing data and limited patient-reported outcomes [79]. Stopa et al. validated a machine learning algorithm for predicting nonroutine discharge after spinal surgery, but other algorithms should be considered for direct comparisons [80]. Huang et al. developed a computer vision algorithm for classifying anterior cervical fusion systems, but further validation and exploration are needed [81]. The utilization of AI in the classification of spine diseases has been extensive. Still, the focus lies in using AI to simulate surgical procedures for enhanced surgical planning and outcome visualization. Key considerations in spinal surgery involve determining the optimal decompression and fixation extent, accounting for the dynamic nature of the spine and the potential risks associated with excessive fixation. Tailoring individualized surgical plans is essential. Retrospective data collection on AI implementation and examination of durotomy and vascular injury cases offer valuable insights. At the same time, robotic surgery adoption faces cost challenges that could be addressed through AI-driven multicenter data analysis. Postoperative care necessitates optimal analgesic use and effective discharge planning, with the potential for ChatGPT to bridge the gap in patient self-observation and engagement in this context.

## 4. Epilepsy

AI has been utilized for predicting the outcome of epilepsy surgery since 1998 [11,82,83,84,85,86,87,88,89]. In one of these early investigations, Grigsby et al. developed a simulated neural network (SNN) to predict seizure-free outcomes after anterior temporal lobectomy using model data from 87 patients [82]. They determined that SNN was superior to a discriminant function in its ability to predict Class 1 (completely seizure-free) and Class 1 or Class 2 (almost or totally seizure-free). The accuracy of the SNNs was 81.3% vs. 78.5% and 95.4% vs. 72.7%, respectively. The retrospective design using patient records and the need for prospective validation with new patients, as well as the potential inclusion of additional input variables such as SPECT and PET, are acknowledged as limitations; however, the study results indicate that simulated neural networks have potential as decision-making adjuncts in epilepsy surgery.

Torlay et al. utilized a language network analysis to classify epilepsy patients based on pre-operative fMRI data [90]. To address this issue, the Extreme Gradient Boosting (XGBoost) technique was employed on five language areas (three frontal and two temporal) activated by fMRI for phonological (PHONO) and semantic (SEM) language tasks. The study, which included 135 patients, found that the subset of left frontotemporal activation caused by the SEM task could distinguish two groups (healthy/typical vs. epilepsy/atypical) with the highest accuracy (AUC of 91 ± 5%).

Meanwhile, Memarian et al. conducted a study on predicting post-surgical outcomes in complicated cases of mesial temporal lobe epilepsy [89]. This retrospective study employed supervised ML to predict postsurgical seizure independence in drug-resistant focal seizures of temporal origin. The study included 20 preoperative patients; these individuals were diagnosed with mesial temporal lobe epilepsy (MTLE) and subsequently underwent the standard procedure of anteromesial temporal lobectomy. The results showed that a combination of maximum relevance minimal redundancy (mRMR) and LA-SVM classifier predicted surgical outcomes with 95% accuracy in atypical mesial temporal lobe epilepsy. The limited spatial coverage of depth electrodes in intracranial EEG recordings poses a constraint, as they are not consistently implanted in all brain areas among patients, but the study’s findings, regarding a higher number of contacts at seizure onset and greater seizures in the ipsilateral amygdala, support the efficacy of amygdalohippocampectomy for achieving seizure freedom in this patient population.

Some groups have leveraged bigger data sets for powerful clinical applications. Abbasi et al. used ML to improve epilepsy diagnosis and therapy by prediction of pharmaceutical response, medical and surgical outcomes, and seizure detection from EEG video and kinetic data [91]. The study highlights the limitations of machine learning techniques in epilepsy, particularly the lack of external validation studies, and emphasizes the importance of larger datasets, cloud-based repositories, and robust external validation to improve the generalizability and interpretability of machine learning models for enhanced clinician confidence and integration into clinical practice. Hosseini et al. studied epileptogenicity locations using Multimodal rs-fMRI and EEG [92]. The study was divided into three phases. First, autonomic edge computing was used to process patient data for determining surgical candidacy. Next, EEG and rs-MRI were used to predict epileptogenic networks. Finally, an unsupervised model-based electrocorticography (ECoG) signals were created to separate interictal epileptic discharge (IED) periods from non-IED periods. The study highlights the limitation of current computational algorithms in reliably identifying preictal periods for effective intervention in epilepsy, emphasizing the need for an autonomic method that accurately detects and localizes epileptogenicity to enhance seizure control and improve quality of life. Using this information as feedback, the authors aimed to improve upon responsive neurostimulation (RNS; Neurospace) management of epilepsy patients.

Temporal lobe epilepsy (TLE) is the most prevalent form of drug-resistant epilepsy in adults [93]. In a noteworthy study by Larivière et al., a multimodal MRI investigation was conducted on 30 drug-resistant TLE patients, utilizing a supervised machine learning approach with fivefold cross-validation [94]. When comparing TLE patients to normal subjects, the findings revealed decreased connectivity distance within the Temporoinsular and Prefrontal networks. Notably, imaging data from patients who underwent anterior temporal lobectomy for seizure treatment and were followed up for one year exhibited an accuracy of 76±4%. While this accuracy may not yet meet translational standards, it marks a significant step forward. This study presents a captivating narrative concerning epilepsy surgery and opens up possibilities for leveraging past data to gain novel insights across multiple centers. However, due to the small training set size, there may be potential bias in patient selection, which is a factor that should be considered in many ML applications in less frequent conditions. The study encountered limitations in sample size, but regularization techniques were used, and the classifier’s performance was compared to a baseline model; however, variability in follow-up times and lack of generalizability to other types of drug-resistant focal epilepsies require further investigation, with initiatives like ENIGMA-Epilepsy being valuable for data coordination, while the openly available surface-based features used in the study can facilitate validation and dissemination.

### Current Challenges

Grisby et al. demonstrate the potential of simulated neural networks (SNN) as decision-making tools for patient selection in epilepsy surgery, while acknowledging the need for further validation and prospective studies [82]. Torlay et al. show promising results in identifying language patterns in epilepsy patients using functional MRI and the Extreme Gradient Boosting algorithm, but call for further research and discussion on the limitations [90]. Abbasi et al. highlight the progress and potential of machine learning in epilepsy but note the lack of critical analysis of challenges and limitations [91]. Hosseini et al. propose autonomic edge computing for epilepsy monitoring but acknowledge the limitations of current computational algorithms in identifying preictal periods accurately [92]. Memarian et al. demonstrate the accuracy of supervised machine learning in predicting postsurgical outcomes for temporal lobe epilepsy, emphasizing the need for additional features [89]. Larivière et al. explore functional and structural changes in epilepsy using machine learning and suggest the role of connectivity distance contractions in personalized surgical prognostication, while acknowledging sample size limitations and the need for further investigation [94].

Epilepsy surgery holds significant importance, but its complexity and limited patient population pose challenges to acquire an accurate diagnosis. Integrating AI as a screening tool and diagnostic aid in epilepsy shows great potential for improving precision and patient care. Furthermore, virtual reality (VR) technology in epilepsy surgery allows surgeons to visualize and simulate procedures with enhanced accuracy and safety. While previous AI research on epilepsy surgery outcomes has provided valuable insights, modern ML applications with larger sample sizes, multicenter data collection, and modern algorithm designs, with mitigation of potential biases, will be necessary before translation to the epilepsy domain.

## 5. Vascular

Artificial intelligence has been widely used in diagnostic imaging to detect cerebrovascular lesions [95,96,97]. Park et al. have used DL to diagnose cerebral aneurysms [97]. The objective of their research was to develop a neural network segmentation model called the “HeadXnet Model” for the prediction of intracranial aneurysms from computed tomography angiography (CTA) data. To achieve this, they utilized a training dataset comprising 611 head CTA data to generate accurate aneurysm segmentation. The model was then evaluated by radiologists using 115 test cases. The study was conducted at one academic medical center where the model was trained, validated, and tested on 818 CTA examinations from 662 patients. Among these cases, 328 were diagnosed with cerebral aneurysms (40.1%), while 490 were negative (59.9%), with the exclusion of cases involving hemorrhage, ruptured aneurysms, arteriovenous malformations, surgical clips, coils, catheters, or other surgical devices. This study’s findings revealed noteworthy improvements among clinicians in various performance measures. The mean sensitivity demonstrated a significant increase of 0.059 (95% CI, 0.028–0.091; adjusted *p* = 0.01), while the mean accuracy exhibited a notable increase of 0.038 (95% CI, 0.014–0.062; adjusted *p* = 0.02). Furthermore, the mean interrater agreement (Fleiss κ) displayed a considerable enhancement, rising from 0.799 to 0.859 with an increase of 0.060 (adjusted *p* = 0.05). Conversely, there was no statistically significant change observed in mean specificity, with an increase of 0.016 (95% CI, −0.010 to 0.041; adjusted *p* = 0.16), or in the meantime to diagnosis, with a difference of 5.71 s (95% CI, 7.22–18.63 s; adjusted *p* = 0.19). The study has several limitations, including the exclusion of ruptured aneurysms and aneurysms associated with other conditions, uncertainty regarding the model’s performance in the presence of surgical hardware or devices, potential interpretation bias due to the high prevalence of aneurysms in the test set and the binary task of clinicians, and limited generalizability of the findings to institutions with different imaging protocols and equipment, as the study was conducted using data from a single institution.

Silva et al. conducted a study utilizing ML techniques to investigate clinical features for detecting aneurysm rupture [98]. They employed three models: RF, linear SVM, and radial basis function kernel SVM. The analysis encompassed 845 aneurysms in 615 patients, of which 309 were classified as ruptured aneurysms. Among the ruptured aneurysms, 307 exhibited aneurysm rupture, accounting for approximately 37% of the study population. The findings revealed that ruptured aneurysms were larger and more commonly located in the posterior circulation than unruptured aneurysms. The ML models achieved AUC values of 0.77 for linear SVM, 0.78 for radial basis function kernel SVM, and 0.81 for the random forest model. The study demonstrated the ability of these ML models to predict aneurysm rupture based on factors such as size and location, with posterior, anterior, and posterior inferior cerebellar arteries frequently associated with aneurysm rupture. Conversely, Paraclinoid and middle cerebral arteries showed a lower likelihood of rupture. These findings align with previous research highlighting the strong correlation between aneurysm location, size, and the risk of rupture. Overall, this study underscores the effectiveness of ML in analyzing complex and extensive data within the field of cerebrovascular neurosurgery, identifying location and size as significant predictors of aneurysm rupture. The study has limitations including the single-institution nature of the patient cohort, the need to assess model performance on external data, the retrospective nature of the data comparing ruptured and unruptured cases, and the lack of long-term follow-up data on untreated aneurysms, suggesting that prospective validation and inclusion of additional radiographic characteristics would be valuable.

Predicting the stability of aneurysms is very useful, especially for small aneurysms [99,100,101,102]. Liu et al. utilized machine learning and radiomics-derived morphological features to predict aneurysm stability, specifically focusing on small aneurysms [103]. The LASSO regression identified several significant morphological features for predicting aneurysm stability. Flatness emerged as the most crucial feature, followed by spherical disproportion, maximum 2D diameter slice, and surface area. Notably, surface area (odds ratio [OR], 0.697; 95% CI, 0.476–0.998), spherical disproportion (OR, 1.730; 95% CI, 1.143–2.658), flatness (OR, 0.584; 95% CI, 0.374–0.894), hyperlipemia (OR, 2.410; 95% CI, 1.029–5.721), multiplicity (OR, 0.182; 95% CI, 0.082–0.380), location of middle cerebral artery (OR, 0.359; 95% CI, 0.134–0.902), and internal carotid artery (OR, 0.087; 95% CI, 0.030–0.211) were included in the final prediction model. The model exhibited commendable performance, with an AUC of 0.853 (95% CI, 0.767–0.940).

Regarding unstable aneurysms, patients with hypertension demonstrated lower values for compactness (*p* = 0.035), sphericity (*p* = 0.035), and flatness (*p* = 0.010), while spherical disproportion (*p* = 0.034) was found to be higher. This showcases the potential of ML in enhancing our understanding and management of aneurysms, especially for smaller aneurysms. QingLin et al.’s work contributes valuable insights to the field of aneurysm stability prediction and emphasizes the role of advanced algorithms in improving clinical decision-making and patient care. This study’s limitations include its single-center nature, the reliance on post-rupture morphology as a surrogate for rupture risk evaluation, the potential misclassification of unstable aneurysms in the absence of definite symptoms, and the limited focus on aneurysms within a specific size range, impeding analysis of smaller aneurysms.

Aneurysmal subarachnoid hemorrhage (aSAH) is frequently linked to high-mortality rates and unfavorable neurological outcomes [104,105,106,107]. In an effort to shed light on prognostic indicators, Koch et al. employed the elastic net (EN) regression and orthogonal partial least squares-discriminant analysis (OPLS-DA) to investigate cerebrospinal fluid (CSF) metabolites associated with poor outcomes [108]. Through the analysis of CSF samples from 81 aSAH patients, 138 metabolites were measured and quantified in each sample. The study revealed the significance of certain vasoactive molecules within the nitric oxide pathway, namely symmetric dimethylarginine (SDMA), dimethylguanidine valeric acid (DMGV), and ornithine, which is associated with poor mRS at discharge (*p* = 0.0005, 0.002, and 0.0001, respectively) and at 90 d (*p* = 0.0036, 0.0001, and 0.004, respectively). Furthermore, in comparison to the nonaneurysmal subarachnoid hemorrhage controls, SDMA exhibited a notably higher concentration in the cerebrospinal fluid (CSF), signifying a significant elevation (*p* = 0.0087). These metabolites were identified as robust predictors of poor outcomes following severe aSAH. This finding offers valuable insights into the underlying molecular mechanisms and provides a potential avenue for prognostic assessment in patients with aSAH. The biased patient cohort limited the retrospective study, lack of correlation between metabolite levels and vasospasm, moderate effect sizes, and potential changes in metabolite profiles over time. Nonetheless, the study further supports the role of symmetric dimethylarginine (SDMA) in subarachnoid hemorrhage (SAH) pathophysiology, highlighting the importance of targeting secondary injury. The unique insight provided by cerebrospinal fluid (CSF) analysis near the site of injury, and whether these metabolites are markers or causative agents, requires further investigation.

Delayed cerebral ischemia (DCI) represents a significant and detrimental complication associated with aneurysmal subarachnoid hemorrhage (aSAH) [109]. To enhance prediction accuracy, Ramos et al. employed ML algorithms that incorporated clinical data combinations with imaging data to forecast the occurrence of DCI [110]. The study, conducted on a cohort of 317 patients, demonstrated that utilizing ML algorithms (e.g., LR, SVM, RFC and MLP) significantly improved DCI prediction. The best AUC of the logistic regression models was 0.63 (95% ci 0.62 to 0.63). The ML algorithms with clinical data improved the AUC to 0.68 (95% ci 0.65 to 0.69). ML models incorporating clinical data and image features achieved the highest AUC values, reaching 0.74 (95% CI 0.72 to 0.75). These findings indicate the potential of ML in augmenting the accuracy of DCI prediction for patients with aSAH, highlighting the value of integrating clinical and imaging data in the prediction process. A limitation of the LR model used in the study is the low number of events per feature, making it prone to overfitting, while the ML algorithms employed can handle high-dimensional feature spaces with less risk of overfitting, but still require external validation; furthermore, determining the best parameter configurations for the ML models can be computationally expensive, and interpreting the 3D image features is challenging, indicating the need for future research in alternative feature extraction techniques for better visualization and interpretation.

In addition to aneurysms, AI has been employed to investigate the factors influencing brain arteriovenous malformation following endovascular embolization, including imaging and clinical presentation to predict procedure complication and outcomes [111]. The study comprised a cohort of 199 participants who underwent brain arteriovenous malformation (BAVM) treatment, with an average follow-up duration of 63 months. The results demonstrated that the standard regression analysis model demonstrated an accuracy of 43% in predicting the outcome (mortality), with the predictor being the overlap of treatment. In contrast, ML exhibited a remarkable accuracy of 97% in outcome prediction of mortality, identifying the presence or absence of a nidal fistula as the most significant factor, irrespective of blinding. Machine learning algorithms have limitations, including their dependency on large training datasets for improved performance and accuracy, the challenge of uncovering the true underlying relationships between factors, the risk of overfitting with irrelevant data, and the need for techniques like cross-validation or regularization to optimize performance and prevent random errors.

Microvascular anastomosis, a surgical procedure that demands exceptional skill, represents a significant clinical challenge. Mastery of this technique requires extensive training, dedication, and persistence. In light of this, Gonzalez-Romo et al. conducted a comprehensive investigation into hand motion during microvascular anastomosis, utilizing a CNN to track 21 hand positions [112]. The study involved six participants, including two experts, two intermediates, and two novices, with no physical constraints imposed on their hand movements. During the subsequent 600-s stimulation period, four non-experts performed 26 anastomotic bites, with an average excess motion of 14.3 (15.5) seconds per bite. In contrast, the expert group completed 33 bites (18 and 15, respectively), exhibiting a mean (SD) excess motion of 2.8 (2.3) seconds per bite for the dominant hand. Additionally, within a 180 s timeframe, the experts accomplished 13 bites, with mean (SD) latencies of 22.2 (4.4) and 23.4 (10.1) seconds, while the intermediate group achieved 9 bites, displaying mean (SD) latencies of 31.5 (7.1) and 23.4 (22.1) seconds per bite. These findings present an intriguing contribution to the field. Although the study featured a relatively small number of participants, the implications are noteworthy, as the results can serve as a valuable resource for future endeavors. This research has the potential to significantly benefit aspiring young neurosurgeons embarking on their journey in microvascular anastomosis, providing them with an opportunity to assess and enhance their skills by comparing their performance to that of experts. Limitations of their study include a small sample size, absence of prospective follow-up, limited assessment of other technique domains, unclear understanding of the relationship between motion analysis and learning curves using different simulators, and the need for further validation and application of the hand detector in clinical settings and with established assessment scales.

### Current Challenges

In a series of studies, researchers utilized various machine learning (ML) techniques to enhance the prediction and understanding of different aspects of aneurysms and cerebral vascular conditions. Park et al. found that integrating the HeadXNet neural network model can enhance clinician performance in detecting intracranial aneurysms, but limitations include the exclusion of ruptured aneurysms, uncertainty regarding the model’s performance with surgical hardware, potential interpretation bias, and limited generalizability to other institutions [97]. Silva et al. demonstrated that machine learning models effectively differentiate between ruptured and unruptured aneurysms based on location and size, but limitations include the single-institution nature of the study and the need for external validation [98]. Liu et al. developed a machine learning model to predict aneurysm stability based on morphological features, but limitations include the single-center nature of the study and the focus on post-rupture morphology [103]. Koch et al. identified metabolites associated with poor outcomes in aneurysmal subarachnoid hemorrhage using machine learning, but limitations include the biased patient cohort and the need for further investigation [108]. Ramos et al. showed improved prediction of delayed cerebral ischemia using machine learning algorithms, but limitations include the moderate predictive accuracy and the need for external validation [110]. Asadi et al. demonstrated the superiority of machine learning in predicting outcomes for brain arteriovenous malformations, but limitations include the dependency on large training datasets and the risk of overfitting [111]. Gonzalez-Romo et al. developed a machine learning-based hand motion detector for microvascular anastomosis simulation, but limitations include the small sample size and the need for further validation and clinical application [112].

The application of AI In aneurysm detection and monitoring has shown promise, although more comparable information is needed for Cavernoma classification. Machine learning (ML) can potentially classify conditions like arteriovenous malformations (AVMs). Complex treatment planning for procedures like clip aneurysms with bypass requires collaboration across departments, and simulating treatment plans using AI for success assessment could be valuable. Integrating AI to capture and compare hand motions between experts and beginners can accelerate skill development and potentially lead to AI-assisted surgical coaching. While molecular-level outcome monitoring has begun, post-surgical care and surveillance require improvement, with limited information available for vasospasm prevention and hypotension monitoring.

The applications of ML to neurosurgery in tumor, spine, epilepsy, and vascular subdomains that were discussed above are summarized in Table 1. There are also currently several interesting clinical trials that have been sourced from clinical trials registered in ClinicalTrials.gov. Most of these trials focus on the diagnostic tests for glioma, followed by clinical trials on aneurysm, which were observational studies presented in Table 2.

## 6. Future Directions

AI has already demonstrated its potential in various aspects of neurosurgery, such as surgical planning, navigation, and image analysis. Looking into the future, AI is expected to play an increasingly significant role in neurosurgery, potentially revolutionizing the field. First, as a precision medicine tool, it can assist neurosurgeons in developing personalized treatment plans. By analyzing an extensive cohort of patient data, medical records, imaging, and genomics, ML can identify patterns that predict treatment response for individual patients. Second, AI supports surgical planning and navigation. Patient imaging can be processed to enable more accurate surgical guidance and real-time feedback during procedures, thereby reducing operative errors. Third, AI enhances the efficiency and accuracy of large data processing, thereby improving diagnoses or uncovering novel therapies. Finally, AI has many important implications for medical education, providing new means of accessing data repositories, such as operative videos to personalize learning and enhanced patient education.

**Figure 4:**
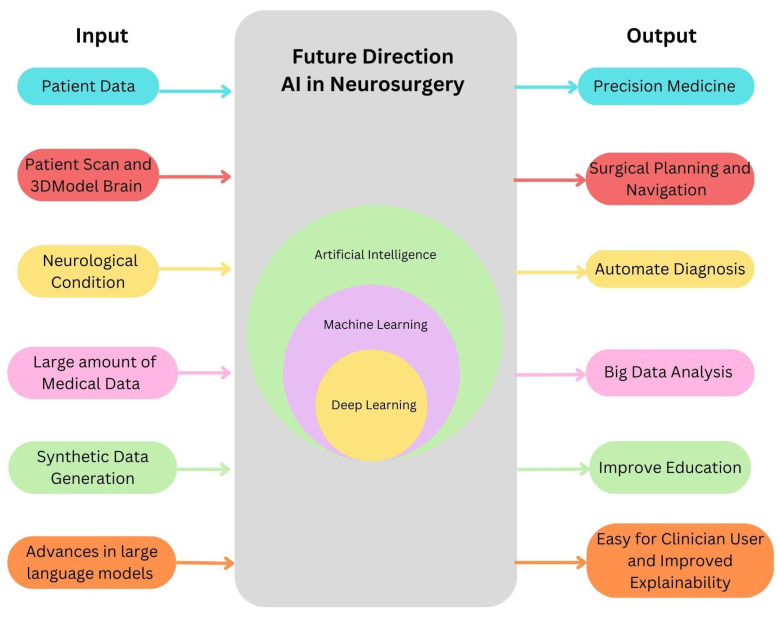
**Figure 4** Future direction AI in neurosurgery.

The recent rise of generative AI has the potential to catalyze AI for neurosurgery in multiple ways. First, generative AI can synthesize new data making training possible for rare conditions and allow for sharing of such synthetic data across centers for multiple-center dataset designs. Second, generative foundational models represent a massive increase in the ability to understand longitudinal multimodal patient data from the patient record and incorporate this data into current predictive models for outcome prediction, surgical planning, and decision–making support. Lastly, large language models like GPT-4 may facilitate ease of use for both clinicians and patients. Clinicians may interact with AI research assisted by GPT-4 code generation as prompted in plain English by clinicians, and automated dataset analysis. Patients can benefit from these models, as such language generation can support physician communication with patients, where patient education is tailored to the individual. One particular use of large language models is that of transparency, where the models can be queried as to what data in their training set was used to make a certain prediction, which may both solve the current ‘black box’ problem as well as allow for active limitation of biases as AI is translated to neurosurgical practice. Based on the information presented above, Figure 4 succinctly encapsulates the forthcoming trajectory of AI in the field of neurosurgery.

## 7. Limitations

Alongside future successes, it is crucial to acknowledge potential challenges that the field may face. One obstacle is a lack of translation and scalability, as clinicians do not widely adopt many AI models in neurosurgery and lack external replication and validation. Additionally, the regulations surrounding AI in healthcare are unclear, and the absence of mandated representation of different backgrounds, such as ethnicities and races in training sets, may perpetuate biases observed in other fields like drug development. Defining AI as a software medical tool in patient care is necessary. Another barrier is limited generalizability of findings, particularly to medically underserved and marginalized groups. This is partially caused by a limited ability to obtain or share data across institutions, creating non-representative training sets. These necessitate a conversation on the potential for similar regulations for AI models as those imposed for clinical trials in drug development (subject cohort design, adverse reporting, etc.…). Finally, as technology progresses beyond ANNs to large language models (LLMs), the cost of training these tools increases. While industry funding may be necessary, the involvement of academia in the development of AI for neurosurgery tools should be considered, and the implications of proprietary AI tools in the field should be examined.

## 8. Conclusions

Recent studies have demonstrated the potential of ML in various aspects of neuro-oncological care, including tumor identification and classification, differential diagnosis, segmentation, molecular classification, personalized anatomical models, intraoperative diagnosis, and survival prediction. ML techniques have also shown promise in spine surgery, predicting adverse events, mortality rates, adjacent segment disease, and various surgical outcomes. In epilepsy, ML has been utilized for predicting seizure outcomes, classifying patients based on fMRI data, predicting postsurgical seizure independence, determining surgical candidacy, and investigating drug-resistant cases. Additionally, ML has been applied to aneurysm prediction, stability assessment, metabolite identification, cerebral ischemia prediction, brain arteriovenous malformation outcome prediction, and microvascular surgery skill assessment. These studies highlight the broad impact and potential of ML in improving patient management and outcomes in neurology and neurosurgery.

In light of what has been stated above, AI is becoming an increasingly common tool in neurosurgery. We provide a synopsis of the primary translational research by dividing the field into four vital neurosurgical sections: tumor, spine, epilepsy, and vascular. As the level of complexity in AI continues to rise, it is vital for us to have knowledge of AI and to be aware of how to maximize its benefits for patient care. Such understanding is essential in order to comprehend the recommendations for application and to be able to anticipate the ongoing trend toward employing AI in the future. This technological understanding will allow us to provide better care for patients, beginning with the diagnosis and counseling, continuing through the planning and procedure, and extending into the post-operative period.

## Figures and Tables

**Table 1:** Studies evaluating machine learning algorithms used for neurosurgical outcome prediction.

1st Author Paper, Year	Output	Input	Output Measures	ML Model	Number of Enrollment	Model Performance	Limitation
Tumor							
Buchlak et al., 2021 [22]	Disease Diagnosis, Outcome	Glioma MRI data	AUC, Sensitivity, Specificity, Accuracy	CNN, SVM, RF	153	AUC = 0.87 ± 0.09 Sensitivity = 0.87 ± 0.10; Specificity = 0.0.86 ± 0.10; Precision = 0.88 ± 0.11	- Large sample size influences NLP classification models. - Conference papers were excluded from the review. - Optimized deep language models are suggested for improved performance. - Readers are referred to specific papers for further information.
McAvoy et al., 2021 [23]	Disease Diagnosis	GBM and PCNSL MRI data	AUC	CNN	320	AUC = 0.94 (95% CI: 0.91–0.97) for GBM AUC = 0.95 (95% CI: 0.92–0.98) for PCNL.	- Retrospective design with a small number of patients from two academic institutions. - The findings may have limited generalizability to other settings. - The use of PNG exports of DICOM images results in data loss. - There is no direct comparison between the classification outcomes of CNNs and radiologists. - Further research is needed to determine the clinical value of the tool.
Boaro et al., 2021 [24]	Automatically segment meningiomas from MRI scan	Meningioma MRI data	Dice score, Hausdorff distance, Inter-expert variability	3D-CNN	806	Dice score of 85.2% (mean Hausdorff = 8.8 mm; mean average Hausdorff distance = 0.4) Median of 88.2% (median Hausdorff = 5.0 mm; median average Hausdorff distance = 0.2 mm) Inter-expert variability in segmenting the same tumors with means ranging from 80.0 to 90.3%	- Limited in its ability to evaluate post-operative residuals, tumor recurrence, or tumor growth due to the inclusion of single pre-operative scans. - Model’s detection performance was not tested on brain MRI scans without meningioma. - Algorithm has not been integrated into the hospital informatics system.
Zhou et al., 2019 [25]	IDH genotype and 1p19q codeletion in gliomas	Preoperative MRI of glioma patients	AUC, Accuracy	ML, RF	538	IDH AUC training 0.921, validate 0.919 Accuracy 78.2%	-Retrospective design and focuses specifically on known gliomas. - Limiting its applicability to different tumor types and non - tumor mimickers.
Tonutti et al., 2017 [32]	Tumor deformation	Load-driven FEM simulations of tumor	Accuracy, Specificity	ANN, SVR	-	ANN model Predicting the position of the nodes with errors <0.3 mm SVR models positional errors < 0.2 mm	- Use of generic mechanical parameters and exclusion of certain brain structures
Shen et al., 2021 [33]	Intraoperative glioma diagnosis	Fluorescence of glioma tissue	AUC, Sensitivity, Specificity	FL-CNN	1874	AUC = 0.945 FL-CNN higher Sensitivity 93.8% vs. 82.0%, *p* < 0.001) Predict grade and Ki-67 level (AUC 0.810 and 0.625)	- Reliance on NIR-II fluorescence imaging. - While NIR-II offers advantages over NIR-I, it may still have lower specificity compared to clinically available methods
Hollon et al., 2021 [34]	Diagnose glioma molecular classes intraoperatively	Raman spectroscopy, coherent anti-Stokes Raman scattering (CARS) microscopy, Stimulated Raman histology (SRH)	Accuracy	CNN	-	accuracy of 92% sensitivity = 93% specificity = 91%	
Tewarie et al., 2022 [40]	Predict outcomes of LMD patients in Brain Metastasis	Clinical Characteristic patient in Brain Metastasis	Risk ratio, *p* value	Conditional survival forest, a Cox proportional hazards model, Extreme gradient boosting (XGBoost), Extra trees, LR, Synthetic Minority Oversampling Technique (SMOTE)	1054	XGboost AUC = 0.83 RFand Cox proportional hazards model C-index = 0.76	The study includes limitations such as a wide time span for patient inclusion. - Including lymph node metastasis as an LMD risk factor is novel and requires more investigation. - Patients receiving only radiation therapy were excluded from the study. - Use of SMOTE reduced data variability. - LMD prognostication at brain metastases (BM) diagnosis is theoretical and not yet widely used in clinical care.
Hulsbergen et al., 2022 [44]	Predicts 6-month survival after neurosurgical resection for BM	Data of Brain Metastasis patient	AUC, Calibration, Brier score	Gradient boosting, K-nearest neighbors, LR, NB, RF, SVM	1062	AUC of 0.71 predicted both 6-month and longitudinal overall survival (*p* < 0.0005)	- Use of retrospective data for internal validation. - The study focuses on survival at a 6-month cutoff rather than overall median survival. - Intraoperative and postoperative factors can influence survival prediction.
Senders et al., 2018 [49]	Predict Survival in GBM patients	Demographic, Socioeconomic, Radiographical, Therapeutic Characteristics	C-index	AFT, Boosted decision trees survival, CPHR, RF, recursive partitioning algorithms	20,821	C-index = 0.70	- Being restricted to continuous and binary models. - Unable to compute subject-level survival curves and lacks interpretability. - Computational inefficiency - Evaluating models based on multiple criteria - Factors unrelated to prediction performance.
Chang et al., 2019 [52]	Evaluation of treatment response	Preoperative MRI of low- or high-grade gliomas, Postoperative MRI with newly diagnosed glioblastoma	Sørensen–Dice coefficient, Sensitivity, Specificity, Dunnet’s test, Spearman’s rank correlation coefficient, intraclass correlation coefficient (ICC)	Deep Learning, Hybrid Watershed Algorithm, Robust Learning-Based Brain Extraction, Brain ExtractionTool, 3dSkullStrip, Brain Surface Extractor	843 preopMRIs from 843 patients with gliomas 713 longitudinal postop MRI from 54 patients with newly diagnosed glioblastomas	Comparing manually and automatically derived longitudinal changes in tumor burden were 0.917, 0.966, and 0.850	- Patient cohort is small and from a single institution. - Lack of comparison with other approaches. - Smaller tumors were excluded from the study. - Variability in MR imaging availability. - Confidence assessment in segmentations is absent
Senders et al., 2018 [53]	Presurgical planning, Intraoperative guidance, Neurophysiological monitoring, and Neurosurgical outcome prediction	Neurosurgical treatment	Median accuracy Dice similarity Median sensitivity coefficient	ANN SVMFuzzy C-means Bayesian Learning RFQuadratic discriminant analysis LDA Gaussian mixture models LR, K-nearest neighbor, NLP K-means	6402	Brain tumor Median Accuracy = 92% Dice similarity coefficient = 88% Radiological of critical/target brain median Accuracy = 94% Dice similarity coefficient = 91% Predict epileptogenic focus Median Accuracy = 86% Detect seizure by iEEG Median Sensitivity = 96% Intraop tumor demarcation Median Accuracy = 89%	- Need for more detailed analysis of all studies and a focus on perioperative care applications. - Caution is advised when interpreting the quantitative performance summary.
Spine							
Fatima et al., 2020 [62]	Clinical decision-making, Patient outcomes	Gender, age, American Society of Anesthesiologists grade, Autogenous iliac bone graft, Instrumented fusion, Levels of surgery, Surgical approach, Functional status, Preoperative serum albumin (g/dL), Serum alkaline phosphatase (IU/mL)	Discrimination, Calibration, Brier score, Decision analysis	LRand LASSO	3965	AUC = 0.7 Brier score = 0.08 Predicting overall AEs Logistic regression = 0.70 (95% CI, 0.62–0.74) LASSO = 0.65 (95% CI, 0.61–0.69)	-Variation in patient and surgical characteristics within the database used. - Limited postoperative outcome data beyond 30 days - Potential missing variables and coding errors in the data are additional limitations.
Karhade et al., 2019 [63]	Postoperative outcome	Preoperative prognostic factor	Discrimination (c-statistic), Calibration (assessed by calibration slope and intercept), Brier score, Decision analysis	SVM, NeuralNetwork (NN)	1790	SVM0.760 NNwith c-statistic 0.769.	- Variable data veracity. - Limited availability of pertinent predictors - Unable to capture the overall trajectory of metastatic disease - lack of explanatory capability. - No examination of multivariate logistic regression or proportional hazard models.
Ames et al., 2019 [64]	Predict surgical outcome	Patient, Surgical factor	*p*-Value	Unsupervised hierarchical clustering	570	overall *p*-value 0.004	- Dependency on sample size - Observation heterogeneity for determining patient and operative clusters.
Goedmakers et al., 2021 [70]	Predicting Adjacent Segment Disease (ASD)	Preoperative Cervical MRI	Accuracy, Sensitivity, Specificity, PPV, NPV, F1-score, Matthew correlation coefficient, Informedness, Markedness	VGGNet19, Resnet18, Resnet50	344	Predict ASD Accuracy = 95% Sensitivity = 80% Specificity = 97%	- Reliance on the last available follow up. - Clinical and demographic characteristics were not considered in the analysis. - Variability in surgical techniques and outcomes. - Small number of MRI scans limited the study. - Distribution of ASD cases were imbalanced.
Karhade et al., 2020 [71]	Incidental durotomies in free-text operative notes	operative notes of patients undergoing lumbar spine surgery	AUC-ROC, Precision-recall curve, Brier score	NLP	1000	AUC-ROC = 0.99 Sensitivity = 0.89 Specificity = 0.99 PPV = 0.89 NPV = 0.99.	- Retrospective nature within a single healthcare system - Influence of shared surgical practices on documentation could affect the results. - Unrecognized or unrecorded incidental durotomies may have been overlooked. - Impracticality of multiple reviews by different researchers or spine surgeons is a limitation of the current work.
Karhade et al., 2021 [72]	Intraoperative vascular injury	age, male sex, body mass index, diabetes, L4-L5 exposure, and infection-related surgery (discitis, osteomyelitis)	C-statstic, Sensitivity, Specificity, PPV, NPV, F1-score	NLP	1035	C-statistic = 0.92 Sensitivity 0.86 Specificity = 0.93 PPV = 0.51 NPV = 0.99 F1-score of 0.64.	- Retrospective design from a single healthcare entity. - Prospective and multi-institutional validation is needed to confirm the findings. - Lack of a rigorous gold standard for intraoperative vascular injury is a limitation. - NLP algorithm used in the study may be prone to overfitting
Karhade et al., 2019 [79]	Prediction of prolonged opioid prescription after surgery for lumbar disc herniation	Chart review of patients undergoing surgery for lumbar disc herniation	C-statistic or AUC, Calibration, Brier Score	Elastic-net penalizedLR, RF, Stochastic Gradient Boosting, NN, SVM	5413	C-statistic = 0.81 AUC 0.81 calibration (slope = 1.13,intercept = 0.13) overall performance (Brier = 0.064)	- Unavailability of opioid dose data and exclusion of illicit opioid use. - Opioid use approximation was based on medical record data - Patient-reported outcomes were not included in the study. - Changing surgical techniques over the study period could have influenced the results. - The study included a limited diversity of institutions.
Stopa et al., 2019 [80]	Nonroutine discharge	Age, Sex, BMI, ASA class, Preoperative functional status, Number of fusion levels, Comorbidities, Preoperative laboratory findings, Discharge disposition	AUC, Discrimination (c-statistic), Calibration, and Positive and Negative predictive values (PPVs and NPVs)	Python (version 3.6) and the R programming language (version 3.5.1).	144	AUC 0.89, calibration slope = 1.09, calibration intercept = −0.08. PPV = 0.50NPV = 0.97.	- Positive findings in terms of external validation. - Different algorithms have shown varying levels of performance in discrimination and calibration.
Huang et al., 2019 [81]	Identification of implanted spinal hardware	AP film cervical radiography after ACDF	Cross-validation analysis Accurracy	KAZE feature detector K-means clustering MATLAB software Vision System Toolbox and Statistics and Machine Learning Toolbox	321	Top choice 91.5% ± 3.8% 2 choice 97.1% ± 2.0% 3 choice 98.4% ± 1.3%	- Limited number of available hardware systems for training. - Additional datasets are needed to evaluate visual artifacts and overlapping radiopaque “noise.” - Prospective data is required to assess the clinical utility of the model. - Potential applications of hardware classification beyond revision ACDF surgery.
Epilepsy							
Grisby et al., 1998 [82]	Predict seizure outcomes	History, Demographics, Clinical examination, Routine scalp EEG, Video-scalp EEG monitoring, Intracranial EEG monitoring, Intracarotid amobarbital (Wada) testing, CT, MRI, Neuropsychological assessment	Accuracy	SNN	87	Accuracy = 81.3% and 95.4%	- Retrospective design with patient records - Prospective validation with new patients is needed for further validation
Torlay et al., 2017 [90]	Atypical language patterns Differentiate patients with epilepsy from healthy people	fMRI	AUC	ML, XGBOOST	55	AUC = 91 ± 5%	
Hosseini et al., 2017 [92]	Epilepsy Seizure Localization	Electroencephalography (EEG), Resting state-functional Magnetic Resonance Imaging (rs-fMRI), Diffusion Tensor Imaging (DTI)	Multiple t-test, Differential connectivity graph (DCG)	CNN	9	*p*-value Normal 1.85 × 10^-14^ Seizure 4.64 × 10 ^-27^	- limitations in reliably identifying preictal periods. - Need for an autonomic method that accurately detects and localizes epileptogenicity.
Memarian et al., 2015 [89]	Predict surgery outcome	Clinical, Electrophysiological, Structural magnetic resonance imaging (MRI) features	Accuracy	LDA, NB, SVM with radial basis function kernel (SVM-rbf), SVM with multilayer perceptron kernel (SVM-mlp), Least-Square SVM (LS-SVM).	20	Accuracy = 95%	- The limited spatial coverage of depth electrodes in intracranial EEG recordings poses a constraint. - Depth electrodes are not consistently implanted in all brain areas among patients.
Larivière et al., 2020 [94]	Predict postsurgical seizure outcome	Multimodal MRI imaging	Accuracy	Supervised machine learning with fivefold cross-validation	30	Accuracy = 76± 4%	- Limitations in sample size. - Regularization techniques were used- Variability in follow-up times and lack of generalizability to other types of drug-resistant focal epilepsies
Vascular							
Park et al., 2019 [97]	Clinician performance with and without model augmentation	CTA examinations	Sensitivity, Specificity, Accuracy, time, interrater agreement	CNN	818	mean Sensitivity increased = 95%, mean Accuracy increased = 95%, mean Interrater agreement (Fleiss κ) increased = 0.060, from 0.799 to 0.859 (adjusted *p* = 0.05) mean Specificity = 95% Time to Diagnosis 95%	- Exclusion of ruptured aneurysms and aneurysms associated with other conditions. - Performance of the model in the presence of surgical hardware or devices remains uncertain. - Potential interpretation bias may exist - Conducted using data from a single institution.
Silva et al., 2019 [98]	Clinical Features, Detection of Aneurysm Rupture	Vascular imaging data of cerebral aneurysms	*p* value, AUC, Sensitivity, Specificity, PPV, NPV	RF, Linear SVM, Radial basis function kernel SVM	845	AUC Linear SVM = 0.77 Radial basis function kernel SVM = 0.78	- Single institution for the patient cohort - The retrospective nature of the data comparing ruptured and unruptured cases is a limitation. - Long-term follow-up data on untreated aneurysms is lacking, which affects the analysis.
Liu et al., 2019 [103]	Predicting Aneurysm Stability	Morphological feature aneurysm	*p* value, Odds ratio, AUC, chi square test, t test	Lasso regression	1139	Flatness (OR, 0.584; 95% CI, 0.374–0.894) Spherical Disproportion (OR, 1.730; 95% CI, 1.143–2.658) SurfaceArea (OR) = 0.697 (95% CI, 0.476–0.998) AUC = 0.853 (95% CI, 0.767–0.940)	- Single-center nature - Reliance on post-rupture morphology as a surrogate for rupture risk evaluation - Potential misclassification of unstable aneurysms without definite symptoms - Limited focus on aneurysms within a specific size range, hindering analysis of smaller aneurysms.
Koch et al., 2021 [108]	Vasoactive molecule that predict poor outcome	CSF of aSAH patients	*p* value 2-tailed student *t*-test, Fischer’s exact test	Elastic net (EN) ML, Orthogonal partial least squares- (OPLS-DA)	138	Poor mRS At Discharge (*p* = 0.0005, 0.002, and 0.0001) At 90 day (*p* = 0.0036, 0.0001, and 0.004)	Biased patient cohort. - No correlation found between metabolite levels and vasospasm. - Effect sizes observed were moderate. - Possibility of changes in metabolite profiles over time.
Ramos et al., 2019 [110]	Prediction of Delay Cerebral Ischemia	Clinical and CT image data	AUC,	LR, SVM, RFMLP, Stock Convolutional Denoising Auto-encoder, PCA	317	Logistic regression models AUC = 0.63 (95% CI 0.62 to 0.63) ML with clinical data AUC = 0.68 (95% CI 0.65 to 0.69) ML with clinical data and image feature AUC = 0.74 (95% CI 0.72 to 0.75)	- LR model used in the study had a limitation of a low number of events per feature, making it prone to overfitting. - ML algorithms used in the study can handle high-dimensional feature spaces with less risk of overfitting but still require external validation. - Determining the best parameter configurations for ML models can be computationally expensive.
Asadi et al., 2016 [111]	Outcome variables, Clinical outcome prediction	Study documented imaging, Clinical presentation, Procedure, complications, Outcomes	Accuracy	Supervised Machine learning MATLAB Neural Network Toolbox	199	Accuracy = 97.5%	- ML algorithms depend on large training datasets for improved performance and accuracy. - Uncovering the true underlying relationships between factors can be challenging for ML algorithms. - There is a risk of overfitting when irrelevant data is included in the training process.
Gonzalez-Romo et al., 2023 [112]	Microvascular anastomosis hand motion	21 tracking hand landmarks from 6 participant	Mean (SD), One-way ANOVA	Python programming language and Mediapipe; CNN	6	6oo s 4 nonexpert 26 bites total 2 expert 33 bites(18 bites and 15 bites) 180 s Expert, 13 bites with mean latencies of 22.2(4.4) and 23.4 (10.1) seconds 2 intermediate, 9 bites with mean latencies of 31.5(7.1) and 34.4 (22.1) seconds per bites	- Small sample size. - Prospective follow up was not conducted. - Assessment of other technique domains was limited. - The relationship between motion analysis and learning curves using different simulators is not well understood.

**Table 2:** Clinical trials on artificial intelligence in neurosurgery.

Trial or Registry	Number Enroll	Condition	Interventions	Outcome Measure	Status
NCT04671368	141	Central Nervous System Neoplasms	Diagnostic Test: Artificial Intelligence Diagnostic Test: Practicing Pathologists Diagnostic Test: Gold Standard	Diagnostic Accuracy of Study Arms Sensitivity and specificity of Study Arms Spearman Coefficient of Study Arms related to Gold Standard	Unknown status
NCT04220424	500	Glioma	Diagnostic Test: MR and Histopathology images based prediction of molecular pathology and patient survival	AUC of Prediction performance	Unknown status
NCT04216550	600	Recurrent Glioma	Drug: Apatinib	Changes of Response to Treatment Progression-Free Survival (PFS) Overall Survival (OS) Incidence of treatment-related adverse events	Recruiting
NCT04215211	2500	Glioma	Diagnostic Test: Survival prediction for glioma patients	AUC of survival prediction performance	Recruiting
NCT04217018	3000	Glioma	Diagnostic Test: Prediction of molecular pathology	AUC of prediction performance	Recruiting
NCT04215224	3500	Glioma	Diagnostic Test: Histopathology images based survival prediction for glioma patients	AUC of survival prediction performance	Recruiting
NCT04217044	3000	Glioma	Diagnostic Test: Histopathology images based prediction of molecular pathology	AUC of prediction performance	Recruiting
NCT04872842	1000	Intracranial Aneurysm	Other: Obervation	Aneurysm rupture Aneurysm growth	Completed
NCT05608122	1000	Intracranial Aneurysm	Other: Obervation	Aneurysm rupture Aneurysm growth	Recruiting
NCT04733638	500	Intracerebral Hemorrhage	Device: Viz ICH VOLUME	Algorithm Performance Algorithm Processing Time Time to Notification Time to Treatment Length of Stay In Hospital Complications Modified Rankin Scale (mRS) at Discharge and 90 Days	Enrolling by invitation
NCT05804474	1500	Intracranial Aneurysm	Other: Observational study	Intracranial aneurysm size Intracranial aneurysm volume Intracranial aneurysm height Intracranial aneurysm neck diameter Parent artery diameter Intracranial aneurysm width Aspect ratio Size ratio	Completed
NCT04608617	1000	Stroke, Ischemic	Device: Viz LVO (De Novo Number DEN170073)	Transfer patients: Time from spoke CT/CTA to door-out Non-transfer patients: Time from Hub door to groin puncture Time from Spoke Door-In to Door-Out (DIDO) Time from Spoke CT/CTA to Specialist Notification Time from Spoke CT/CTA to Groin Puncture Time from Spoke Door to Groin Puncture Length of ICU Stay/Total Length of Stay Modified Rankin Scale (mRS) at Discharge and 90 Days National Institutes of Health Stroke Scale (NIHSS) at Discharge Patient Disposition at Discharge and 90 Days	Recruiting
NCT05099627	300	Cervical Myelopathy	Other: The questionnaire “Cervical myelopathy treatment outcome questionnaire” Other: The retrospective questionnaire “CSM early diagnosis questionnaire” Other: JOACMEQ questionnaire and mJOA	JOACMEQ questionnaire mJOA questionnaire Cervical myelopathy treatment outcome questionnaire” CSM early diagnosis questionnaire” Nurick score DOR (diagnostic odds ratio)	Not yet recruiting
NCT05161130	1115	Low Back Pain Disk Herniated Lumbar Spinal Stenosis Lumbar	Procedure: Lumbar Spinal Fusion	Visual Analogue Scale for Back Pain Visual Analogue Scale for Leg Pain Oswestry Disability Index	Completed
NCT04359745	500	Glioblastoma		Accuracy of the artificial intelligence model Failure rate of the artificial intelligence model	Recruiting
NCT04467489	1040	Cerebral Cavernous Malformation Cavernous Angioma Hemorrhagic Microangiopathy	Other: observational	Circulating Diagnostic and Prognostic Biomarkers of CASH Correlation of Imaging and Plasma Biomarkers of CASH Confounders of CASH Biomarkers	Recruiting
NCT04819074	4000	Aneurysm, Brain	Procedure: Microsurgery	modified Rankin Scale Sensorimotor neurological deficits Clavien Dindo Complication Grading	Recruiting

## Data Availability

Not applicable.
